# Patterns and Geographical Mechanism of Altitudinal Belts in Tropical African Mountains

**DOI:** 10.1002/ece3.72426

**Published:** 2025-10-30

**Authors:** Jiayu Li, Baiping Zhang, Yonghui Yao, Ya Jiang, Junjie Liu

**Affiliations:** ^1^ State Key Laboratory of Resources and Environmental Information System Institute of Geographic Sciences and Natural Resources Research Beijing China; ^2^ University of Chinese Academy of Sciences Beijing China

**Keywords:** altitudinal belt spectrum structure, aspect differentiation, climatic factors, combination patterns, tropical African Mountains, vertical range

## Abstract

Altitudinal belts exhibit substantial variation across the world's mountains in number, typology, combination patterns, and vertical range. However, the conditions under which specific belts occur and the climatic factors influencing their vertical range remain poorly understood. Therefore, this study focuses on tropical African mountains, which are characterized by massive volcanic cones, rich biodiversity, and complete altitudinal belt structure, as a representative region. We compiled 23 spectra of altitudinal belts from published literature for 10 representative tropical African mountains. Integrating climatic data of WorldClim V2.0 and topographic data from SRTM 90 m DEM, we investigated the vertical ranges and combination structures of altitudinal belts, and analyzed their relevant driving climatic factors using stepwise regression. The results show that: (1) Tropical African mountains usually have five to eight altitudinal belts which constitute a complete altitudinal belt spectrum from tropical vegetation to nival zones. (2) The upper montane regions are typically characterized by the development of bamboo forest, cloud forest, and ericaceous forest belts, but with different patterns of single belt, dual belts and triple belts. (3) Climate‐altitudinal belt regression models could well explain the vertical range of the highest forest belts (ericaceous forest) and low forest belt (monsoon rainforest) (*R* ^2^ = 0.72–0.75), and could moderately explain the vertical range of mountain forest belt, bamboo forest belt and cloud forest belt (*R* ^2^ = 0.31–0.44). (4) The normal establishment of a specific altitudinal belt primarily depends on annual hydrothermal conditions or on a compensatory interplay between temperature and precipitation under suboptimal conditions, while the vertical range of any altitudinal belt is closely associated with the intra‐annual or seasonal variations of hydrothermal conditions. This study further highlights the complexity and diversity of tropical African mountains, providing a more solid scientific foundation for altitudinal belt theory development.

## Graphical abstract

Tropical African mountains usually exhibit five to eight altitudinal belts, forming a complete altitudinal belt spectrum from tropical vegetation to nival zones. Our results show that the normal establishment of a specific altitudinal belt primarily depends on annual hydrothermal conditions, while the vertical range of any altitudinal belt is closely associated with the intra‐annual or seasonal variations of hydrothermal conditions.

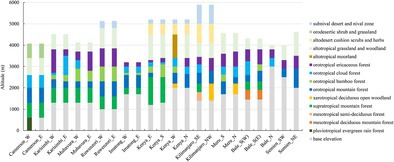

## 1 Introduction

Altitudinal vegetation belts and their vertical combination (spectrum) can well reflect the basic characteristics of high mountains and the relationship between mountain environments and vegetation. They exhibit substantial variation across the world's mountains in number, typology, combination patterns, and vertical range, highlighting the complexity and diversity of mountain altitudinal zonation. The study of the spectral structure of altitudinal belts is a classic paradigm and basic modeling method to reveal the complexity of the mountain environment (Zhang et al. 2003). Since Alexander von Humboldt (1816) first established the scientific classification of altitudinal zonation in the Andes Mountains in the early 19th century, substantial achievements have been made in the investigation of altitudinal zonation, classification of altitudinal belts, and their regional and global patterns and mechanisms (Zhang et al. 2009). However, it remains unclear under what conditions different types of altitudinal belts occur, and what factors determine the vertical range of the same belt type across different mountains.

Africa is the second largest continent in the world, with an area of approximately 30 million km^2^ which accounts for about 20.2% of the world's land area. The East African Rift System (EARS) extends over 3000 km from north to south, traversing the Ethiopian and East African plateaus, with the Western Branch being a younger terrain and the Eastern Branch an older and volcanically active domain (Ring et al. 2018). It encompasses a diverse variety of natural landscapes, ranging from humid tropical rainforests to arid deserts and being global biodiversity hotspots (Mittermeier et al. 2011; Couvreur et al. 2021; CEPF, n.d.). Among these landscapes, the tropical African mountains, which are characterized by massive volcanic cones, rich biodiversity, and a complete altitudinal belt structure, represent an ideal region for investigating the formation mechanisms of altitudinal zonation.

The first systematic classification of East Tropical African mountain vegetation was developed by Hedberg (1951), which identified three principal vegetation belts: montane forest belt, ericaceous belt, and alpine belt, with the montane forest belt being further subdivided into montane forest, bamboo and *Hagenia*‐*Hypericum* zones. Boughey (1956) incorporated data from West Tropical Africa to develop a more flexible and comparative generalized classification scheme. Bussmann (2006) comprehensively reviewed vegetation zonation patterns across African mountain systems and proposed a refined nomenclature for Afrotropical Mountains based on climate and plant community characteristics, providing a more detailed subdivision classification scheme of altitudinal belts, refining Hedberg's forest zones into distinct belts.

In terms of altitudinal zonation and its underlying mechanisms, Friis (1992) and Friis and Lawesson (1993) confirmed the existence of an altitudinal zonation with critical altitudinal limits in the forest flora in northeastern tropical Africa through various clustering methods. Lovett (1996) analyzed the altitudinal and latitudinal variations of tree communities and diversity with different precipitation patterns of three typical mountains in Tanzania's Eastern Arc Mountains. Hemp (2005) identified distinct discontinuities across all strata (trees, shrubs, epiphytes, lianas, and herbs) through one‐dimensional constrained clustering analysis of vegetation transect data from Mt. Kilimanjaro, which were significantly correlated with altitude, temperature, and soil acidity. Ochola (2019) investigated the altitudinal distribution of plant species richness on Mt. Kenya. Lasway et al. (2023) analyzed how annual mean temperature and precipitation drive variations in woody plant species richness, tree height, and floristic composition on the eastern slope of Mt. Meru, revealing that precipitation had a greater impact on low‐altitude plant communities compared to those at higher elevations.

These studies mentioned above have predominantly focused on qualitative descriptions and the nomenclature of vegetation zonation, with limited involvement of spatial patterns of main altitudinal belts and with very limited quantitative analysis of the spectral structure of altitudinal belts across tropical African regions. This study seeks to address these gaps by quantitatively investigating how climatic factors determine the presence and vertical range of specific altitudinal belts, with tropical African mountains serving as a representative case. It aims to illuminate the diversity and complexity of Afrotropical montane forest ecosystems through the novel perspective of altitudinal belt spectrum structures, and provide a more solid scientific foundation for the theoretical development of mountain altitudinal belts.

## 2 Material and Methods

### 2.1 Study Area

**FIGURE 1:**
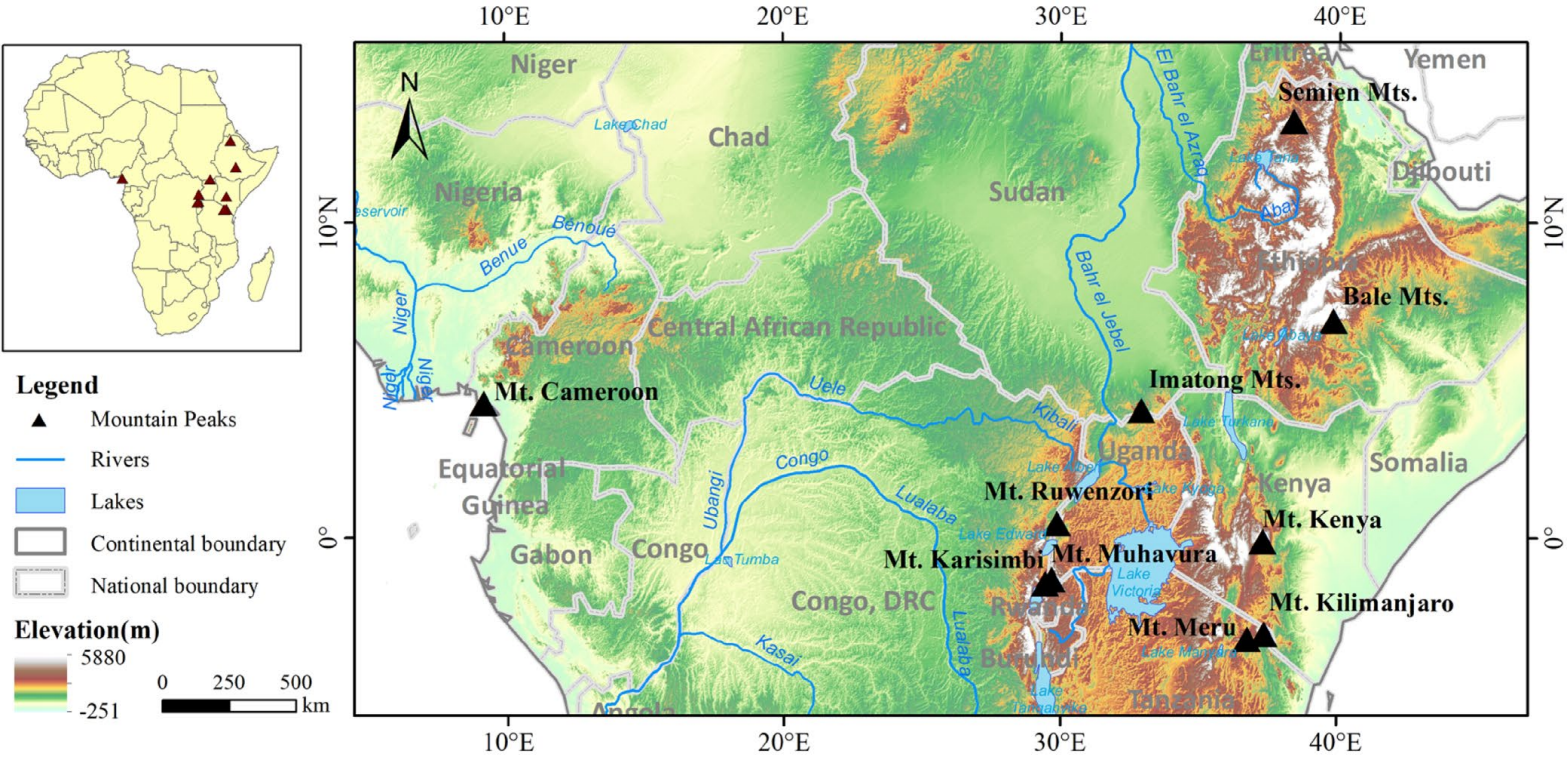
Spatial distribution of typical mountains in tropical Africa.

A total of 10 mountains with distinct altitudinal vegetation differentiation were selected for study, including Mount Cameroon (4070 m) in West Africa, Mount Ruwenzori (5127 m), Mount Karisimbi (4507 m), Mount Muhavura (4127 m) in Central Africa, and Semien Mountains (4620 m), Bale Mountains (4620 m), Imatong Mountains (3200 m), Mount Kenya (5199 m), Mount Kilimanjaro (5895 m), and Mount Meru (4567 m) in East Africa, as shown in Figure 1. These study sites essentially cover the typical mountainous regions of tropical Africa across a spatial gradient from north to south and west to east. Together these mountains span the major climatic gradients of tropical Africa, ranging from humid equatorial landmass with mean annual precipitation exceeding 2500 mm (Mt. Cameroon) to semi‐arid highlands receiving precipitation of less than 800 mm (Semien Mountains), and with dry seasons from 3 to 7 months.

### 2.2 Mountain Altitudinal Belt Data

**FIGURE 2:**
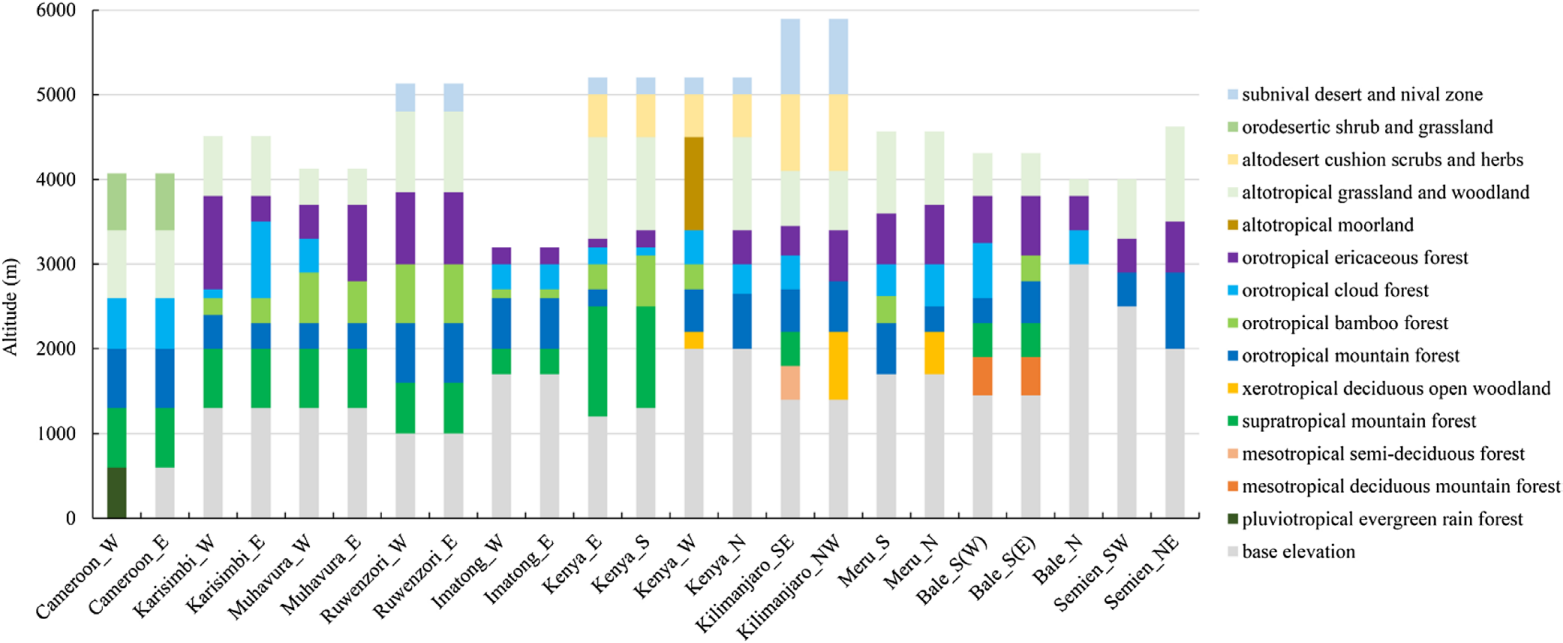
Altitudinal belt spectra of typical mountains in tropical Africa.

Totally 23 altitudinal belt spectra from 10 typical mountains were extracted following the classification framework proposed by Bussmann (2006), adopting his nomenclature of vegetation belts in Afrotropical Mountains (Figure 2). Because Bussmann provided detailed slope‐specific profiles and corresponding elevational ranges of vegetation zonation, no further reclassification or reinterpretation was conducted in this study. Each slope provided by Bussmann was considered an independent spectrum, named according to the mountain and slope orientation (e.g., “Kilimanjaro_NW” and “Kenya_S”). In cases where intra‐slope variations were identified (e.g., a bamboo belt occurring on the eastern part of the southern slope of the Bale Mountains but absent on its western part), these sections were treated as independent altitudinal belt spectra. All elevation data were expressed in meters above sea level.

Following Bussmann's classification, 14 types of altitudinal belts have been identified across these mountains, including nine forest belts, of which half are widely distributed (Figure 2). This study focuses on the five most widespread forest belts that are both ecologically representative and commonly observed across tropical African mountains. The representative tree species for each forest belt are listed in Table S1.

1. Supratropical mountain forest (STMF), also known as tropical montane rainforest (TMMRF), is the main type of altitudinal belt in the lower montane regions. The main vegetation consists of camphor forests dominated by *Ocotea usambarensis*.
2. Orotropical mountain forest (OTMF) is distributed on almost all mountain slopes and is characterized by montane mixed evergreen forest, including two subtypes: humid and dry. The Humid OTMF contains mixed evergreen broad‐leaved, coniferous and sclerophyllous species, whereas the Dry OTMF lacks evergreen broad‐leaved species.
3. Orotropical bamboo forest (OTBF) is one of the endemic altitudinal belts dominated by *Sinarundinaria alpine* (or *Yushania alpina*) in the Afrotropical Mountains, which forms a continuous and distinct bamboo zone at mid‐elevations.
4. Orotropical cloud forest (OTCF) occurs in zones perpetually shrouded in mist, where clouds penetrate the forest canopy and create a humid and dim environment rich in epiphytes (Jarvis and Mulligan 2010). The dominant tree species is *Hagenia abyssinica*, commonly accompanied by *Hypericum revolutum*.
5. Orotropical ericaceous forest (OTEF), sometimes regarded as the treeline ecotone, is the vegetation zone dominated by *Erica* spp., which constitutes the upper limit of African mountain forests.

### 2.3 Topographic and Climatic Data and Processing

1. Topographic data were extracted from SRTM 90 m DEM Version 4 provided by the Consortium for Spatial Information (CGIAR‐CSI) (https://srtm.csi.cgiar.org/srtmdata/), with a spatial resolution of 3 arc sec (approximately 90 m resolution). The boundaries of typical mountain ranges were delineated by the integration of the standardized mountain inventory data from the Global Mountain Biodiversity Assessment (GMBA) (Snethlage et al. 2022a, 2022b), and the terrain ruggedness index derived from DEM data. The area surrounding each mountain peak was divided into eight equal sectors (numbered 1–8 in a clockwise direction), using the peak as the vertex and true north as 0°. For instance, the eastern slope was represented by sectors 2 + 3, while the northwestern slope comprised sectors 7 + 8. According to the aspects and vertical ranges of different types of altitudinal belts in each mountain, we created corresponding masks based on the elevation data and slope aspect classification results.
2. Due to the scarcity of meteorological stations in African mountains and the lack of climate data at different altitudes, we used publicly available, globally interpolated climatological datasets to extract climatic factors within each altitudinal belt. We initially used CHELSA V1.2 due to its theoretical advantages in simulating mountain climates (Karger et al. 2017, 2018; Bobrowski et al. 2021) and its demonstrated consistency with observed precipitation trends on Mt. Kilimanjaro (Appelhans et al. 2016; Hemp 2006). However, a preliminary comparison on Mt. Ruwenzori revealed a discrepancy between CHELSA outputs and published regional descriptions: CHELSA indicates higher precipitation on the eastern slope, whereas Bussmann (2006) reports a relatively moist western slope and a drier eastern slope. Therefore, we ultimately selected WorldClim V2.0 (https://www.worldclim.org/) for subsequent analysis, as it shows precipitation patterns more consistent with existing regional descriptions. The dataset (30 arc sec, approximately 1 km resolution) was resampled to match the resolution of the DEM data using the bilinear interpolation method. Nine variables we selected to represent the basic climatic conditions of the study area are: BIO1 (Annual Mean Temperature, °C), BIO4 (Temperature Seasonality, °C), BIO5 (Max Temperature of Warmest Month, °C), BIO6 (Min Temperature of Coldest Month, °C), BIO7 (Temperature Annual Range (BIO5‐BIO6), °C), BIO12 (Annual Precipitation, mm), BIO15 (Precipitation Seasonality), BIO16 (Precipitation of Wettest Quarter, mm), and BIO17 (Precipitation of Driest Quarter, mm). The arcpy package in Python was used to batch extract these bioclimatic variables according to the masks, and zonal statistics were carried out to calculate the mean values of bioclimatic indicators within each belt.
3. Meteorological data from nine weather stations nearest to each mountain were obtained from the Global Summary of the Day (GSOD) and Global Historical Climatology Network—Daily (GHCN‐Daily) published by the National Centers for Environmental Information (NCEI) of the National Oceanic and Atmospheric Administration (NOAA) (https://www.ncei.noaa.gov/). Monthly and annual precipitation averages were calculated for the period 1981–2020, also obtaining the intra‐annual precipitation patterns and maximum duration of dry seasons (Table S2). Dry seasons mean months with precipitation less than 60 mm according to Köppen's tropical climate classification system as (Kottek et al. 2006).

### 2.4 Statistical Analysis

To investigate the controlling factors of the vertical range of altitudinal belts, Pearson correlation analysis was first conducted to examine the relationships between vertical ranges and bioclimatic variables. Subsequently, stepwise linear regression models were constructed with bioclimatic variables as predictors and the vertical range of each belt as the response variable. The bioclimatic variables were used in their original units without standardization to preserve the interpretability of the results. The assumptions of normality and homoscedasticity were visually confirmed with residual Q‐Q and scatter plots (Appendix S1). All analyses were conducted using IBM SPSS Statistics 26.

## 3 Results

### 3.1 Vertical Range and Controlling Factors of Altitudinal Belts in Tropical African Mountains

#### 3.1.1 Distribution Patterns of Major Altitudinal Belts and Their Vertical Ranges

##### 3.1.1.1 Tropical Montane Monsoon Rainforest Belt

**FIGURE 3:**
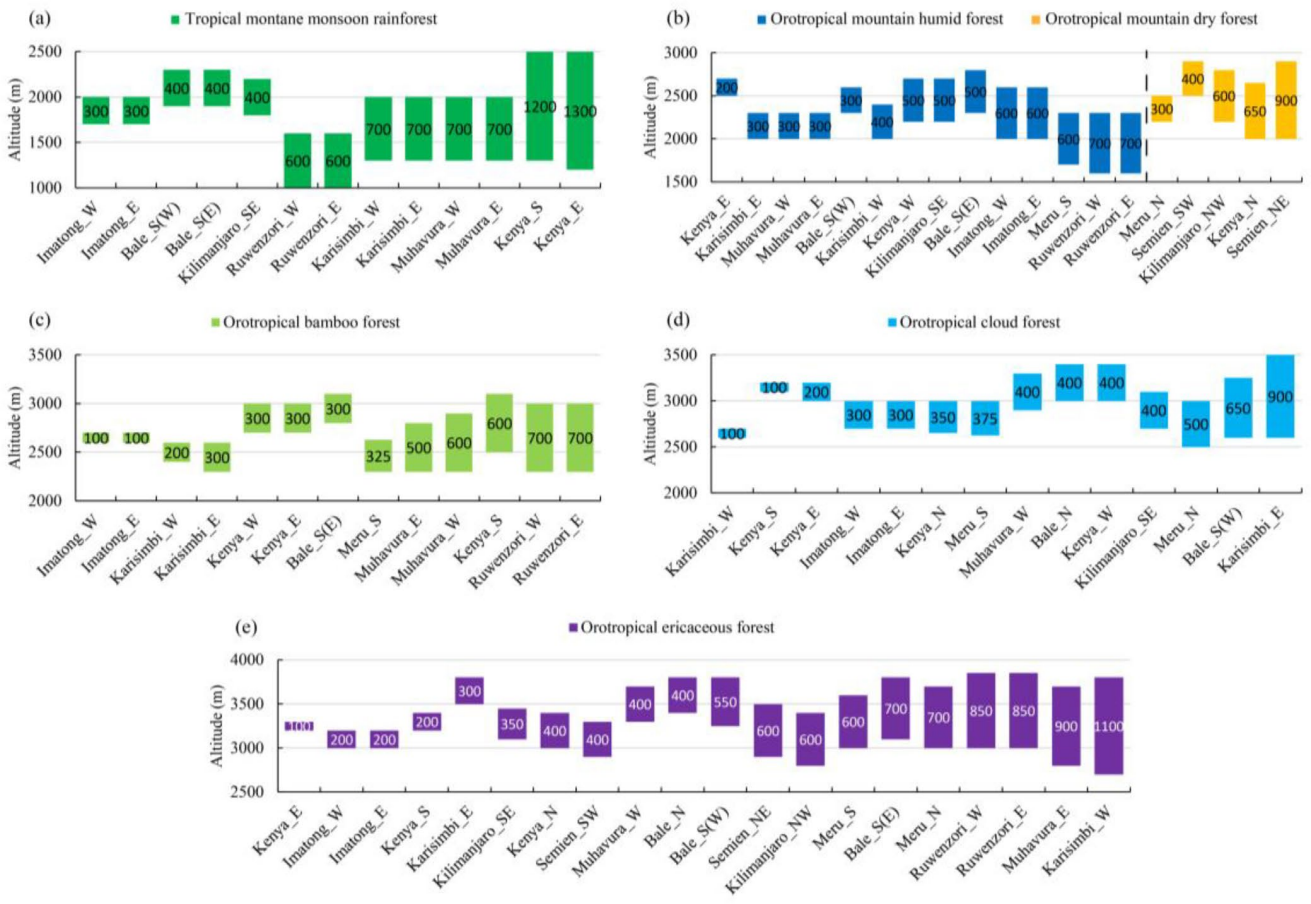
Spatial distribution and vertical range of major altitudinal belts in tropical Africa.

It is the principal basal belt in the study area, with belt widths ranging from 300 m to 1300 m, predominantly between 600 and 700 m (Figure 3a). The widest occurs between 1200 and 2500 m a.s.l. on the eastern slope of Mt. Kenya, which crosses the equator. Its lower part (1200–1500 m) contains such plant species as *Lovoa swynnertonii* (evergreen), *Chrysophyllum gorungosanum* (evergreen), and *Newtonia buchananii* (deciduous), which can all adapt to short‐term drought. The upper section (1500–2500 m) features typical camphor forests. The second widest belt (1200 m) appears on the southern slope of Mt. Kenya and consists exclusively of camphor communities. The narrowest belt (only 300 m) occurs on both eastern and western slopes of the Imatong Mountains, where the base elevation is relatively high (1700 m a.s.l.). The next narrowest belts (400 m) are found above the local basal belts (mesotropical semi‐deciduous forest) on the southern slopes of Mt. Kilimanjaro and above deciduous mountain forest in the Bale Mountains in East Africa.

##### 3.1.1.2 Orotropical Mountain Forest Belt

The vertical range of this belt is from 200 to 900 m, mostly between 500 and 700 m (Figure 3b). Its humid type is widely distributed in the central and western African mountains and on the windward slopes of eastern African mountains. It reaches a vertical range of 700 m in both eastern and western slopes of Mt. Cameroon and Mt. Ruwenzori. The narrowest belt (200 m) occurs on the windward (eastern) slopes of Mt. Kenya; while on the southern slope, humid OTMF is absent and replaced by bamboo forest. The dry OTMF is restricted to the leeward slopes of eastern African mountains and the northeastern Ethiopian Plateau, with widths decreasing from 900 m on the northeastern slope of the Semien Mountains to 300 m on the northern slope of Mt. Meru.

##### 3.1.1.3 Orotropical Bamboo Forest Belt

The bamboo belt is only distributed in the central and eastern African mountains, with a width ranging from 100 to 700 m (Figure 3c). In central Africa, the belt is generally more than 500 m in width, reaching up to 700 m on both the eastern and western slopes of Mt. Ruwenzori. In eastern Africa, its width reaches 600 m on the southern (windward) slope but is absent on the leeward (northern) slope of Mt. Kenya.

##### 3.1.1.4 Orotropical Cloud Forest Belt

This belt appears across all mountain ranges except for Mt. Ruwenzori and the Semien Mountains, with widths varying from 100 to 900 m, predominantly between 300 and 500 m (Figure 3d). On extremely humid Mt. Cameroon in West Africa, the belt maintains the same width of 600 m on both eastern and western slopes. In East Africa, the belt is narrow (100–375 m) on windward slopes and relatively broad on leeward slopes (350–500 m). In the Virunga Mountains of Central Africa, relatively wide cloud forest belts (400–900 m) have developed on the eastern slope of Mt. Karisimbi and the western slope of Mt. Muhavura. In contrast, the belt width is only 100–300 m on the outer slopes of the Virunga Mountains, and completely absent on Mt. Ruwenzori.

##### 3.1.1.5 Orotropical Ericaceous Forest Belt

The ericaceous belt is distributed across all African mountains except Mt. Cameroon in West Africa, with widths ranging from 100 to 1100 m, predominantly between 400 and 700 m (Figure 3e). The widest belts occur in the interlacustrine highlands of Central Africa, namely on the western slope of Mt. Karisimbi. In East Africa, its width is less than 700 m, usually wider on leeward slopes. On the periphery of the East African Plateau, the belt is quite narrow, only 100–350 m, such as on the eastern and western slopes of the Imatong Mountains and on the southeastern slopes of Mt. Kenya and Mt. Kilimanjaro.

#### 3.1.2 Climatic Factors Related With Altitudinal Belts

By relating climatic factors with the occurrence of altitudinal belts, we identified the fundamental conditions required for the formation of each belt (Table 1 and Table S3). Subsequently, stepwise linear regression analysis of belt width against intra‐belt bioclimatic parameters revealed the primary factors that determine the vertical range of each altitudinal belt (Table 1).

**TABLE 1:** Fundamental conditions for the development of major altitudinal belts and their width‐controlling climatic factors in Central and East African Mountains.

Belt type	Bioclimatic variables	Fundamental conditions	Narrow belt conditions	Normal belt conditions		Wide belt conditions	Width‐controlling factors	Model equation	*R* ^2^
Tropical montane monsoon rainforest	BIO1 (°C)	15.3–21.3	19.5–19.8	16.0–16.8	16.7–21.3	15.3–16.9	BIO12	*y* = 1.301*BIO12−1156.037	0.724*** *n* = 13 df = 12
	BIO12 (mm)	1081–1754	1227–1399	1081–1272	1269–1563	1730–1754			
	Belt Width (m)	300–1300	300	400	600–700	1200–1300			
Orotropical mountain forest	BIO1 (°C)	12.8–18.2	13.0	13.8–16.2	13.5–18.2	17.3	BIO5	*y* = 57.268*BIO5−819.906	0.310* *n* = 19 df = 18
	BIO5 (°C)	20.3–27.3	20.3	22–24.3	21.4–25.6	27.3			
	BIO12 (mm)	772.4–1562.5	1467.3	996.4–1562.5	1089.4–1504.8	772.4			
	Belt Width (m)	200–700	200	300–400	600–700	900 m			
Orotropical bamboo forest	BIO1 (°C)	10.9–15.3	15.0°C–15.3°C	10.9–13.2	11.8–14.6	12.9–13.1	BIO17	*y* = 1.810*BIO17 + 116.508	0.406* *n* = 13 df = 12
	BIO7 (°C)	10.7–15.9	12.9°C–13.0°C	13.7–15.9	10.7–13.2	11.5–11.6			
	BIO12 (mm)	1134.9–1675.4	1466–1499	1134.9–1163.5	1265.9–1675.4	1545.1–1589.7			
	BIO17 (mm)	24.3–271.5	83–84	24.3–78.6	108.2–215	249–271.5			
	Belt Width (m)	100–700	100	300–325	500–600	700			
Orotropical cloud forest	BIO1 (°C)	9.5–14.7	9.5–13.3	14.5–14.7	12.9	11.2–12	BIO15/Local Topography	*y* = 5.975*BIO15−67.966 (*n* excludes points mainly affected by local topography)	0.441* *n* = 11 df = 10
	BIO4 (°C)	24–148.4	26–83.2	118.3–119.4	137.0	32.1–58.9			
	BIO12 (mm)	1099.7–1781.8	1444.7–1732	1493.7–1520.2	1234.7	1167.8–1661.1			
	BIO15 (%)	41.4–85	41.8–58.8	62.2–62.2	83.2	41.4–52.5			
	Belt Width (m)	100–500 (900)	100–200	300	500	650–900[T]			
Orotropical ericaceous forest (complete)	BIO1 (°C)	8.1–11.8	8.7–9.7	11.8–11.8	8.6–10.1	9.1–11.4	BIO7&BIO16/BIO12/Local Topography	*y* = −125.299*BIO7−2.737*BIO16 + 3806.070 (*n* excludes points mainly affected by local topography or with BIO12 < 1000 mm)	0.753*** *n* = 13 df = 12
	BIO7 (°C)	9.9–21.1	13.7–15.6	20.2–21.1	12.7–16.8	10.3–11.3			
	BIO12 (mm)	869.2–1811.7	1343.7–1739.6	869.2–966.4	1205.9–1381.6	1459.7–1732			
	BIO16 (mm)	437.7–670.7	538–661.2	606.1–670.7	437.7–590.3	510.7–580.3			
	Belt Width (m)	100–1100	100–350	400–600	600–700	850–1100			

*Note:* ***p < 0.001, *p < 0.05. [T] indicates data points primarily influenced by local topography.

**FIGURE 4:**
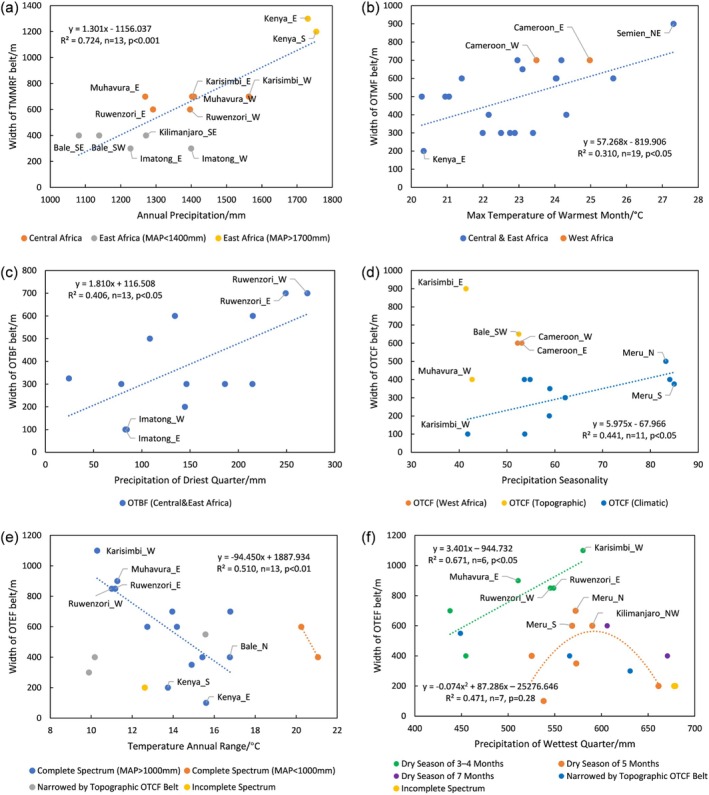
Relationships between (a) the width of the TMMRF belt and annual precipitation; (b) the width of the OTMF belt and the max temperature of the warmest month; (c) the width of the OTBF belt and the precipitation of the driest quarter; (d) the width of the OTCF belt and precipitation seasonality; (e) the width of the OTEF belt and temperature annual range; (f) the width of the OTEF belt and the precipitation of the wettest quarter.

1. The montane monsoon rainforest belt corresponds to a mean annual temperature (MAT) of 15°C–21°C and a mean annual precipitation (MAP) of 1050–1750 mm. In Central Africa with a MAP around 1400 mm and less seasonal climate variations, this belt can develop well with belt widths of 600–700 m. In Eastern Africa, mountains exhibit greater climatic seasonal variability. On the eastern and southern windward slopes of Mt. Kenya, abundant rainfall exceeding more than 1700 mm supports the development of tropical monsoon rainforest even at lower elevations, forming a combined base and typical altitudinal belt with a vertical range of 1200–1300 m. However, this belt becomes significantly narrower (300–400 m) where annual precipitation falls below 1400 mm (Figure 4a). Statistical analysis indicates that this belt shows a highly significant widening trend with increasing annual precipitation (*R* ^2^ = 0.724, *p* < 0.001, *n* = 13).
2. The mountain forest belt typically develops under conditions of MAT between 12.8°C–18.2°C and MAP of 700–1600 mm. In addition, it also develops under exceptionally warm and humid conditions (MAT: 18.2°C–18.9°C, MAP: 2700–2800 mm) on Mt. Cameroon in West Africa, demonstrating its broad ecological adaptability across diverse climatic regimes. Statistical analyses reveal a significant positive correlation between its width and the maximum temperature of the warmest month (*R* ^2^ = 0.31, *p* < 0.05, *n* = 19). For instance, on the northeastern slope of the Semien Mountains, where this belt reaches its maximum width (900 m), the maximum temperature of the warmest month attains 27.3°C. In contrast, on the eastern slope of Mt. Kenya, where the corresponding temperature is lower (20.3°C), the belt narrows markedly to merely 200 m (Figure 4b). A maximum temperature of the warmest month of approximately 20°C appears to represent the lower thermal threshold for the development of OTMF. Furthermore, humid OTMF is extensively distributed on windward slopes of mountains in East Africa with MAP exceeding 1100 mm, and on mountains in Central and West Africa, while dry OTMF predominates on both windward slopes with MAP below 1100 mm and leeward slopes in East Africa, reflecting precipitation‐driven ecological differentiation within this forest type (Figure 3b).
3. The bamboo belt primarily requires MAT of 10.6°C–16°C and MAP of 1400–1700 mm for its normal development. In regions with MAP below 1400 mm but relatively short dry seasons (≤ 4 months), this belt can still persist if the temperature annual range does not exceed 16°C. However, if the dry season lasts for 5 months or more, the temperature annual range must remain below 14°C for the belt to develop. If MAP falls below 1100 mm, this belt fails to develop. Statistical analysis reveals that its width increases significantly with rising precipitation during the driest quarter (*R* ^2^ = 0.406, *p* < 0.05). For example, the belt reaches its maximum width (700 m) on Mt. Ruwenzori with the driest quarter precipitation exceeding 250 mm, whereas it narrows to merely 100 m on the Imatong Mountains with less than 100 mm in the driest quarter (Figure 4c).
4. The cloud forest belt typically develops when MAT attains 9.5°C–14.7°C and MAP reaches 1100–1800 mm. Its distribution is closely associated with persistent mountain fog patterns, so this belt should only be classified as an azonal vegetation belt rather than a typical altitudinal belt. It is virtually related to local orographic structures. For instance, Mt. Karisimbi and Mt. Muhavura, both parts of the semicircular Virunga volcanic chain, are separated by a saddle region at an elevation of approximately 2600 m, which facilitates the accumulation of moist air and the formation of dense cloud masses, contributing greatly to the development of a significantly wider cloud forest belt along the inner slopes of the Virunga Mountains. Notably, the eastern slope of Mt. Karisimbi hosts the widest OTCF belt (900 m) in tropical Africa. Similarly, the 650 m thick cloud forest belt on the western part of the southern slope of the Bale Mountains may be associated with the frequent formation of cloud and mist thanks to the interception of moist southwesterly air currents by the Harenna Escarpment (Hillman 1988; Yirdaw et al. 2015). The vertical thickness of this belt increases significantly with greater precipitation seasonality (*R* ^2^ = 0.441, *p* < 0.05). For instance, the belt reaches its maximum width of 500 m on the southern slope of Mt. Meru, where precipitation seasonality is relatively high (83.2%), but narrows to 100 m on the western slope of Mt. Karisimbi, where precipitation seasonality is lower (41.8%) (Figure 4d). Notably, Mt. Ruwenzori has a high MAP of 1600–1800 mm but a low seasonality (~30%), without the formation of a cloud forest belt, suggesting that the seasonal distribution pattern of precipitation may play a more critical role than MAP in determining the existence of a cloud forest belt.
5. The ericaceous forest belt occurs with a MAT between 8.1°C–11.8°C and MAP 800–1900 mm, reflecting its tolerance to low temperatures and a broad range of precipitation conditions. The upper boundary of the ericaceous belt is actually the local treeline. In the Semien Mountains, where MAP is below 1100 mm, the ericaceous forest is the only forest type present in the upper montane zones, reaching a belt width of 400–600 m. On the inner slopes of the Virunga Mountains and the western part of the southern slope of the Bale Mountains, local orographic effects facilitate the development of broad OTCF belts, which necessarily squeezes the vertical space available for OTEF development to only 300–550 m. In regions where the ericaceous belt develops under normal conditions—that is, where mountain peak elevations exceed the regional climatic treeline and are not significantly affected by local topography or precipitation limitations—the belt width is strongly and negatively correlated with both temperature annual range and precipitation of the wettest quarter (*R* ^2^ = 0.753, *p* < 0.001). What's more, temperature annual range contributes the most to the variation in belt width (63.8%); while the relative contribution of precipitation in the wettest quarter is only 36.2%. Scatterplots (Figure 4e) could further illustrate this pattern: in Central Africa, where the temperature annual range is relatively small (10.3°C–11.3°C), the ericaceous belt can reach widths of 850–1100 m. In contrast, on the windward slopes of Mt. Kenya and Mt. Kilimanjaro with a broader annual temperature range of 13.7°C–15.6°C, the belt is considerably narrow (100–350 m). The ericaceous belt reaches maximum width when the precipitation of the wettest quarter amounts to approximately 550–600 mm (Figure 4f). Both excessive and insufficient wet‐season precipitation lead to a reduction in width or even the disappearance of the belt. For instance, the belt is absent on the western slope of Mt. Kenya, where the precipitation of the wettest quarter exceeds 700 mm, and on Mt. Cameroon, where it approaches 1100 mm.

### 3.2 Bamboo‐Cloud‐Erica Combination Patterns and Occurrence Conditions

**FIGURE 5:**
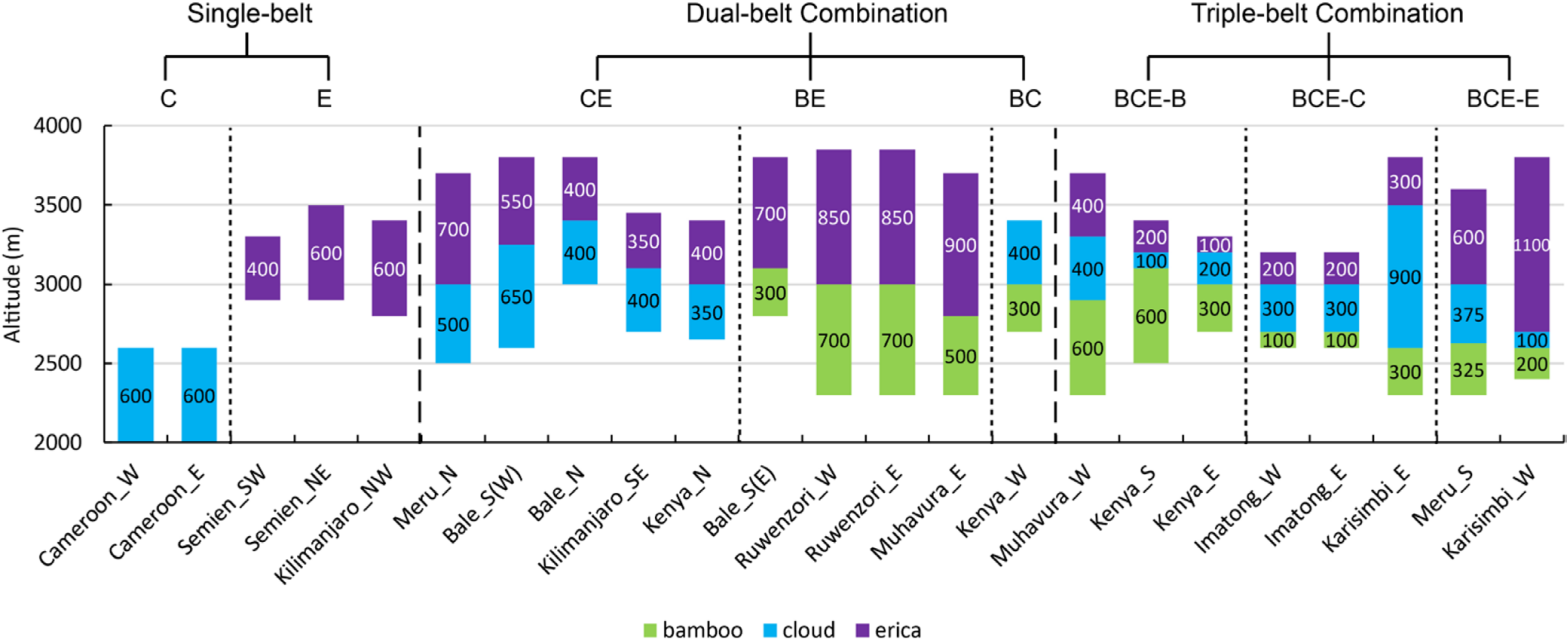
Types and vertical distribution patterns of Bamboo‐Cloud‐Erica (BCE) combinations.

The most characteristic feature of mountain altitudinal belts in tropical Africa is the occurrence of the bamboo forest belt, cloud forest belt, and ericaceous forest belt in various combinations in the upper montane zone, hereafter referred to as the Bamboo‐Cloud‐Erica (BCE) combination. Figure 5 illustrates the vertical distribution patterns of BCE assemblages across different mountains, with the corresponding climatic conditions and geographic locations shown in Table S4.

#### 3.2.1 Single‐Belt (C/E)

The occurrence of the single‐belt pattern is often the result of extreme climatic conditions. For instance, on Mt. Cameroon in West Africa, where MAP exceeds 2600 mm, excessive moisture limits the development of both the OTBF and OTEF belts, allowing only the moisture‐adapted cloud forest belt to dominate the upper montane regions. In contrast, in the Semien Mountains (MAP < 1000 mm) and on the rain‐shadow slopes of Mt. Kilimanjaro, only the ericaceous belt is present with broad ecological adaptability, while the development of bamboo and cloud forests is limited. Notably, the bamboo belt does not occur independently.

#### 3.2.2 Dual‐Belt Combination (CE/BE/BC)

The dual‐belt combination is typically found in areas with generally suitable conditions, but specific climatic limitations prevent the establishment of the third belt. The Cloud‐Erica (CE) combination predominantly occurs in East Africa with MAP of 1100–1400 mm but where the annual temperature range (14°C–16°C) exceeds the upper limit tolerated by bamboo forest. These areas exhibit significant precipitation seasonality (53%–84%) and moderate precipitation of the wettest quarter (450–600 mm), with cloud and ericaceous forest belts in relatively close proportion. The Bamboo‐Erica (BE) combination is primarily found in the Central African Mountains where MAP (1400–1800 mm) is high but precipitation seasonality is low (29%–59%), with the ericaceous belt consistently occupying a larger proportion (55%–70%). The Bamboo‐Cloud (BC) combination only appears on the western slope of Mt. Kenya, where the precipitation for the wettest quarter exceeds 700 mm, and the two belts have a similar width of approximately 300–400 m.

#### 3.2.3 Triple‐Belt Combination (BCE)

The coexistence of all three belts (BCE) typically occurs in regions with relatively high MAP (1400–1800 mm) and moderate precipitation seasonality (40%–60%). This combination can be further categorized into three types based on the dominant belt.

The Bamboo‐dominated combination (BCE‐B): it typically occurs in areas with relatively high precipitation in the driest quarter (134–215 mm), such as the western slope of Mt. Muhavura and the windward (southern and eastern) slope of Mt. Kenya. The bamboo belt has a large proportion in the vertical range of the combination, from 43% to 67%, increasing with precipitation in the driest quarter.

The Cloud‐dominated combination (BCE‐C): it is found in mountain regions with relatively low precipitation of the driest quarter (80–150 mm) but abundant precipitation of the wettest quarter (600–700 mm) in the upper montane zone, such as the eastern and western slopes of the Imatong Mountains and the eastern slope of Mt. Karisimbi. For example, in the Imatong Mountains, where precipitation seasonality exceeds 60%, the width proportion of the cloud forest belt accounts for 50%. On the eastern slope of Mt. Karisimbi, despite its relatively moderate precipitation seasonality (~41%), low dry‐season and high rainy‐season precipitation and favorable local topography together promote cloud formation, resulting in an exceptionally wide cloud forest belt (900 m) which occupies 60% of the BCE combination.

The Erica‐dominated combination (BCE‐E): it appears on mountains with the precipitation of the wettest quarter of 550–600 mm, which has been identified as optimal condition for ericaceous development (see Section 3.1). On the western slope of Mt. Karisimbi, the ericaceous belt reaches the maximum width of 1100 m and the highest proportion of 79% in the BCE combination, indicating a strong ecological advantage. On the southern slope of Mt. Meru, the BCE combination exhibits a structurally balanced pattern, with a width of 300–600 m for each belt. Although the MAP is only about 1200 mm, the small temperature annual range (< 14°C) enables the establishment of the bamboo belt. The high precipitation seasonality (85%) supports the formation of the cloud forest belt, while a suitable amount of precipitation during the wettest quarter (570 mm) promotes the dominance of the ericaceous belt, which accounts for 46% of the BCE combination.

### 3.3 Aspect‐Related Differentiation of Altitudinal Belts

**FIGURE 6:**
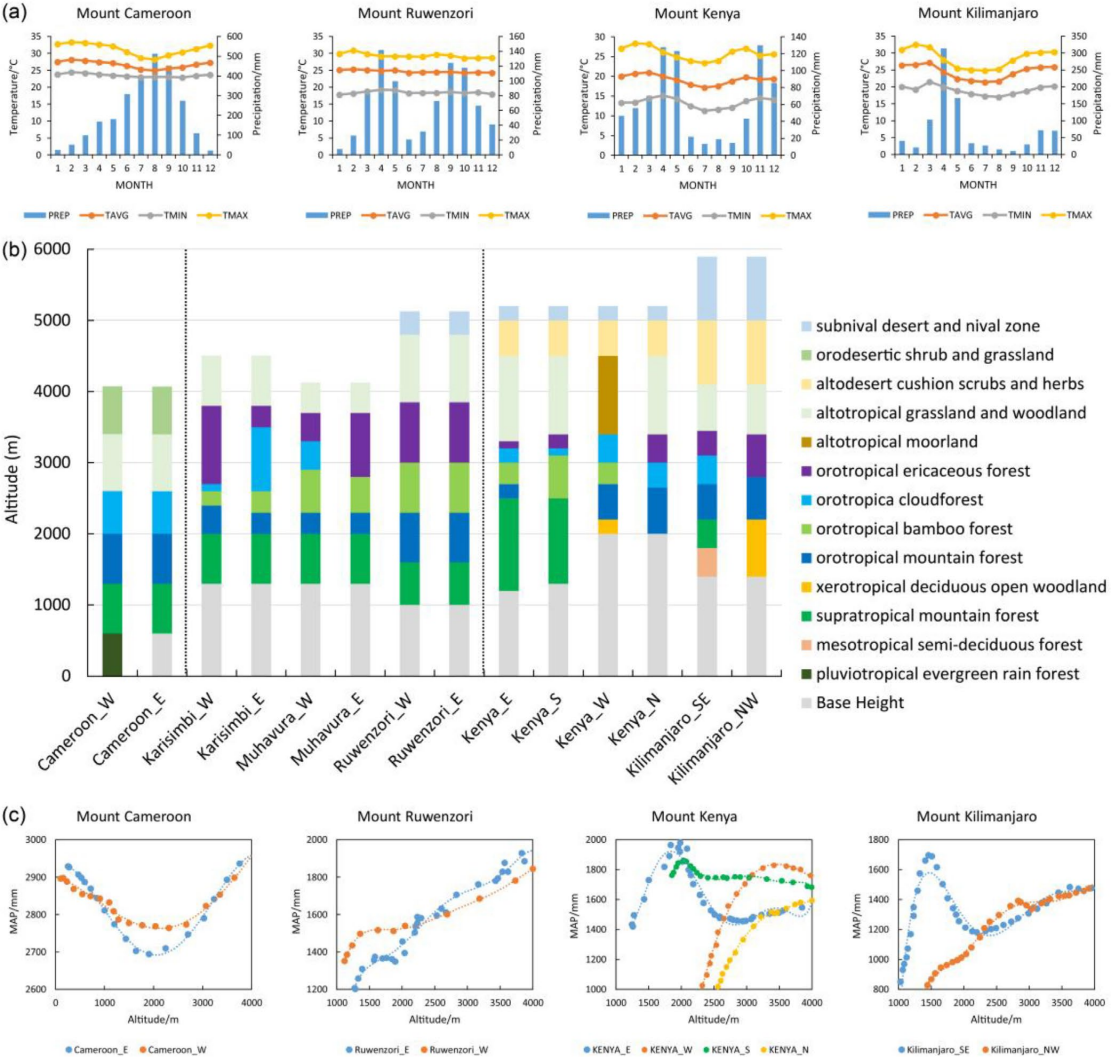
Aspect‐related patterns of precipitation and altitudinal belts in tropical African mountains. (a) Monthly temperature and precipitation patterns at the climate station nearest to each mountain. Climate variables include monthly precipitation (PREP, mm), mean temperature (TAVG, °C), mean maximum temperature (TMAX, °C), and mean minimum temperature (TMIN, °C). (b) Aspect differentiation in the altitudinal belt spectrum of representative tropical African mountains. (c) Altitudinal variation patterns of mean annual precipitation (MAP, mm) along different aspects.

Aspect‐related differences in mountain vegetation zonation are common and complex. This is quite so for African mountains. To characterize such differences, we can explore how moisture condition is associated with local altitudinal belts on different slopes based on meteorological station data near each mountain. Furthermore, vertical profiles of MAP were extracted along the central lines of contrasting aspects. The results are shown in Figure 6.

1. In West Africa, where MAP exceeds 2500 mm, aspect has no discernible effect on vegetation zonation, as is the case of Mt. Cameroon (Figure 6b).
2. In the interlacustrine highlands of Central Africa, there are two rainy seasons of comparable intensity and a relatively short dry season lasting about 3 months. The lower montane zones exhibit little aspect differentiation, while the upper montane zones have varying belt widths due to local orographic conditions. For example, the Virunga Mountains, with their arc‐shaped configuration, show a pronounced climate contrast between the inner and outer slopes. Mt. Karisimbi and Mt. Muhavura are located at opposite ends of the arc, approximately 29 km apart, and share similar regional climatic conditions. The opposing inner slopes (the eastern slope of Mt. Karisimbi and the western slope of Mt. Muhavura) facilitate cloud accumulation and support a well‐developed cloud forest belt (900 m wide), whereas the width of the ericaceous belt remains narrow (200–300 m). In contrast, on the outer slopes (Karisimbi's western slope and Muhavura's eastern slope), the cloud forest belt is nearly absent (0–100 m wide), and the ericaceous belt expands significantly (up to 1100 m wide). Notably, similar to Mt. Cameroon, Mt. Ruwenzori also exhibits no evident aspect differentiation in altitudinal belts. The forest belts on both eastern and western slopes are of comparable width, typically ranging from 600 to 800 m, which may be regarded as an ideal “similar‐width belt structure.”
3. The aspect effect of altitudinal belts is most pronounced in East African Mountains, which are mainly influenced by the southeastern monsoon from the Indian Ocean and characterized by distinct wet and dry seasons. Plant composition is quite different between windward and leeward slopes, usually from broad‐leaved and malacophyllous species to coniferous and sclerophyllous species. Mt. Kenya, which straddles the equator, serves as the most representative example. The altitudinal belts exhibit complex variations with aspect. In terms of altitudinal belt combinations, only the windward slopes (eastern and southern) support a triple‐belt combination (BCE), whereas the Erica belt is absent on the western slope, and the bamboo belt is missing on the northern slope. The bamboo belt is widest (600 m) on the wet southern slope, narrows on the eastern and western slopes (to about 300 m), and disappears on the drier northern slope. In contrast, the cloud belt is broader on the leeward slopes (350–400 m) than on the windward slopes (100–200 m). As for Mt. Kilimanjaro, only a dual‐belt combination develops on the southeastern slope, with no bamboo belt. On the northwestern slope, only the Erica belt remains, with its vertical range increasing from 350 m on the southeastern slope to 600 m on the northwestern. It is evident that slope aspect exerts a significant influence on the number, types, vertical range, and combination patterns of altitudinal belts.
4. Patterns of vertical variation in precipitation further reveal region‐specific trends across tropical African mountains. In Central and West Africa, altitudinal trends of annual precipitation exhibit consistent patterns across different aspects of the same mountain, although the specific patterns differ between these two regions. Specifically, in West African Mountains, precipitation typically decreases with elevation at lower altitudes, then increases at higher elevations. Central African Mountains exhibit a continuous increase in precipitation with elevation across all slopes. East African Mountains display markedly different vertical precipitation patterns between their windward and leeward slopes. On the windward slopes, precipitation commonly peaks in the lower montane zones (typically between 1500 and 2500 m), followed by a gradual stabilization of precipitation with increasing elevation. In contrast, precipitation on the leeward slopes increases rapidly from lower values at the mountain base, and around mid‐elevations (approximately 2300–2500 m), it often approaches or even surpasses that on the windward side at the same altitude.

## 4 Discussion

### 4.1 Reliability and Limitations of Global Climate Datasets

The accuracy of climate data is always an issue to consider in mountain research. Owing to the lack of continuous in situ observations for each altitudinal belt, this study relied on globally interpolated climatological datasets as the analytical foundation. However, it is important to recognize that such datasets may have limitations in certain regions, especially where terrain is highly complex. During data selection (see Section 2.3), we found that CHELSA may underestimate precipitation on the western slope of Mt. Ruwenzori. According to Jury (2024), a lee‐side rotor sustaining rainfall on the Congo Basin side may explain the higher moisture levels on the western slope. This small‐scale topographic process is likely unresolved in CHELSA, leading to biased precipitation estimates. Similar to findings from the Himalayas by Datta et al. (2020), which showed that model performance is sensitive to the choice of climate dataset and may vary regionally, this highlights the importance of careful validation against regional knowledge prior to specific application.

Moreover, as Hemp and Hemp (2024) demonstrated for the Tanzanian mountains, both WorldClim and CHELSA showed significant differences from field observations in terms of annual precipitation and the elevation of peak rainfall. Bioclimatic analyses in northern Patagonia (Fierke et al. 2024) and in northern Peru (Newell et al. 2022) likewise emphasized the limitations and potential biases when using these datasets for precipitation analysis. Therefore, the interpretation of the results should avoid being overly reliant on absolute values and instead focus more on their relative trends and spatial patterns.

### 4.2 On the Explanatory Ability of Climatic Versus Non‐Climatic Factors for Altitudinal Belts

Both climatic and non‐climatic factors have been proposed to influence the distribution of mountain altitudinal belts. While climate is generally considered the dominant driver, the elevational patterns of montane forest belts can be further modified by non‐climatic mechanisms, such as edaphic (Hemp 2005), topographic heterogeneity (Beals 1969; Guo 2014), biotic interactions (Hamilton 1981; Bussmann 2006; Zhang et al. 2022) and disturbance regimes (Wesche et al. 2000; Hemp and Beck 2001). We have noted that the vertical range could be modified by biotic interactions with adjacent belts. On Mt. Kenya, for instance, OTMF and OTBF often exhibit a reciprocal pattern of distribution or mutual compression (Figure 6b). Bussmann (2006) mentioned that in areas with lower rainfall and higher soil temperatures, individual trees such as Podocarpus latifolius, one of the dominant species of the OTMF belt, can emerge through dense bamboo stands, suggesting competition and replacement between these two belts at the species level. Hamilton (1981) also noted that even where bamboo was absent, tree density was markedly reduced, implying the natural competitive dominance of bamboo communities in mid‐elevation mountain forests. Conversely, the formation of the super‐wide montane deciduous broad‐leaved forest belt in the Qinling Mountains demonstrates the opposite effect. There, the rich diversity and strong competitiveness of the dominant deciduous woody plants allow them to drive the expansion of the belt's width (Zhang et al. 2022), showcasing how competitive advantage can also lead to a broader vertical range. Disturbances such as fire and human activity can further influence the actual distribution of altitudinal belts. The occurrence of fire‐tolerant Erica species can likely be attributed to repeated burning resulting from human activities (e.g., using fire to improve grazing conditions) (Wesche et al. 2000), and the replacement and altitudinal position between *Podocarpus* forests and *Ericaceous* forests on Mt. Kilimanjaro is strongly regulated by fire intensity and frequency (Hemp and Beck 2001).

Our results indicate that climatic factors, notably precipitation, significantly influence the existence and vertical range of altitudinal forest belts in tropical African mountains. Our regression analysis showed a high explanatory ability for the highest forest belts (ericaceous forest) and low forest belt (monsoon rainforest) (*R* ^2^ = 0.72–0.75), but only moderate performance for the mid‐elevation belts (*R* ^2^ = 0.31–0.44). Nevertheless, we consider this moderate explanatory power to be ecologically meaningful, as the models were statistically significant (*p* < 0.05) and successfully identified key climatic factors that account for the observed belt variations. The moderate explanatory power for the middle part belts could be attributed to the complex hydrothermal conditions and biotic interactions in the middle part of mountains. Taking OTMF as an example, it persists across a broad precipitation range (MAP of 700–2800 mm) and is characterized by transitional mixed forest. This strong environmental adaptability and transitional character necessarily lead to varying belt widths difficult to capture precisely using single climatic factors.

So, climatic factors can only partially explain the overall trends in the vertical range of altitudinal belts. The actual variations in belt width are, in fact, the result of the interplay between both climatic and non‐climatic factors. Therefore, constructing multifactor models or attempting to incorporate interaction terms between factors could better explain the variations in the vertical range of mountain altitudinal belts in future research.

### 4.3 Prospect for Future Research on Altitudinal Belts

Our study links altitudinal belts to environmental factors so as to explore the variation in vertical ranges and combination of altitudinal belts. Actually, the upper and lower boundaries of any altitudinal belt are not fixed at a specific elevation, and they rather exhibit a mosaic pattern or transitional zones of about 100 m (Zhao et al. 2023). The idealized model cannot fully reflect these details and is therefore only suitable for exploring the general variation trends of altitudinal belts across different mountains. Further exploration of the fine‐scale interactions between vegetation zonation and climate will require support from multisource datasets, including field survey data and high‐resolution remote sensing imagery products. In addition, this study uses average climatic variables for a certain belt to represent climatic conditions, which is simply a simplified and idealized alternative approach. In reality, the relationships between altitudinal belts and zonal and even non‐zonal factors are highly complex. The observed vertical range of an altitudinal belt is already the dynamic equilibrium state formed by the interaction of various factors under specific mountainous conditions. The influencing factors actually include plant physiological traits (e.g., cold and drought tolerance) (Wang 2022), inner structure of altitudinal belts (Liu 2024), mountain base elevation and relative elevation (Li et al. 2023), and the biogeographical context (e.g., a geographic transition zone or ecotone) (Zhang et al. 2022). Future research should incorporate multiple ecological factors and integrated hydrothermal indices to explore further the mechanisms underlying altitudinal belts, so as to promote the development and refinement of the theory of mountain altitudinal belt structure.

## 5 Conclusions

1. Tropical African mountains typically have five to eight altitudinal belts, and their most distinctive feature is the varied combination and pattern of bamboo, cloud, and ericaceous forest belts, reflecting the complexity of the mountain environment and the diversity of altitudinal belts.
2. The establishment of a specific altitudinal belt is primarily determined by fundamental climatic conditions, such as annual mean temperature and precipitation, while the vertical range of altitudinal belts is closely associated with the intra‐annual or seasonal variations of hydrothermal conditions.
3. The close relationship between altitudinal belts and climatic conditions indicates the sensitivity of mountain vegetation to future climate change and the necessity of monitoring dynamics of mountain ecosystems under global warming.

## Author Contributions

**Jiayu Li:** conceptualization (lead), data curation (lead), formal analysis (lead), methodology (lead), supervision (equal), visualization (lead), writing – original draft (lead), writing – review and editing (supporting). **Baiping Zhang:** conceptualization (lead), methodology (lead), supervision (lead), writing – original draft (supporting), writing – review and editing (lead). **Yonghui Yao:** supervision (equal), writing – original draft (supporting), writing – review and editing (supporting). **Ya Jiang:** formal analysis (supporting), supervision (supporting), writing – review and editing (supporting). **Junjie Liu:** formal analysis (supporting), supervision (supporting), writing – review and editing (supporting).

## Conflicts of Interest

The authors declare no conflicts of interest.

## Supporting information


**Table S1:** Representative plant species composition of altitudinal forest belts.xlsx.

**Table S2:** Meteorological data for stations nearest to each mountain.xlsx.

**Table S3:** Fundamental conditions for the development of major altitudinal belts in West African Mountains.xlsx.

**Table S4:** Climatic conditions for the BCE combinations.xlsx.

**Appendix S1:** Results of stepwise regression analysis.docx.

**Appendix S2:** ece372426‐sup‐0006‐AppendixS2.xlsx.

**Appendix S3:** ece372426‐sup‐0007‐AppendixS3.xlsx.

## Data Availability

No new data were generated in this study. All data used were obtained from publicly available sources or compiled from previously published works. Altitudinal belt spectra were compiled and standardized from Bussmann (2006). Topographic data (SRTM 90 m DEM Version 4) were obtained from the Consortium for Spatial Information (CGIAR‐CSI) (https://srtm.csi.cgiar.org/srtmdata/), and mountain range boundaries were derived from the Global Mountain Biodiversity Assessment (GMBA) inventory (Snethlage et al. 2022a, 2022b). Bioclimatic data were obtained from WorldClim v2.0 (https://www.worldclim.org/), and meteorological station data were retrieved from the Global Summary of the Day (GSOD) and Global Historical Climatology Network—Daily (GHCN‐Daily) published by the National Centers for Environmental Information (NCEI) of the National Oceanic and Atmospheric Administration (NOAA) (https://www.ncei.noaa.gov/). The processed dataset used for analysis is provided as a supporting file for peer review.
