# Supplementary material for: Patterns and Geographical Mechanism of Altitudinal Belts in Tropical African Mountains
Source: Ecol Evol. 2025 Oct 30;15(11):e72426. doi: 10.1002/ece3.72426 (PMC12572827; doi:10.1002/ece3.72426)
Supplement: Supplementary file 5 — Appendix S1: Results of stepwise regression analysis.docx. [file ECE3-15-e72426-s001.docx]

**Appendix A**

**TABLE A1** Stepwise linear regression results for the vertical range of the tropical montane monsoon rainforest belt (TMMRF).

| Model | Unstandardized Coefficients | | Standardized Coefficients | t | Sig. | Collinearity Statistics | |
| --- | --- | --- | --- | --- | --- | --- | --- |
|  | B | Std. Error | Beta |  |  | Tolerance | VIF |
| (Constant) | -1156.037 | 337.159 |  | -3.429 | **0.006** |  |  |
| BIO12 | 1.301 | 0.242 | 0.851 | 5.376 | **0.000** | 1.000 | 1.000 |

**Model fit:** n = 13, R² = 0.724, F (1, 11) = 28.902, p = **0.000**, significant at p < 0.05 (bold)


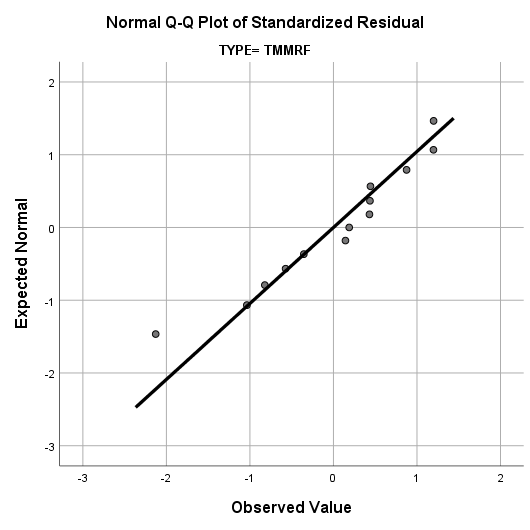


**FIGURE A1** Normal Q-Q Plot of Standardized Residuals for the Regression Model of TMMRF.


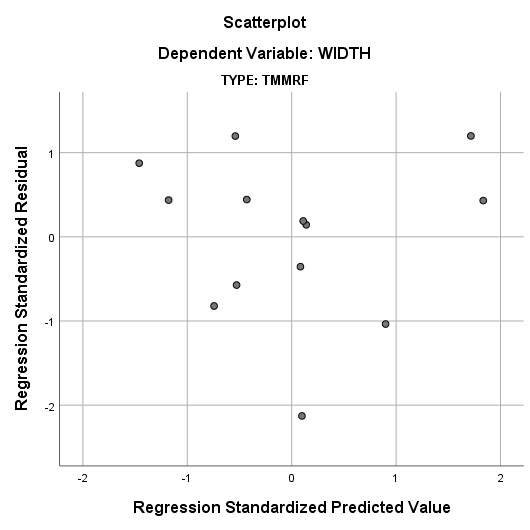


**FIGURE A2** Scatter plot of Standardized Residuals versus Standardized Predicted Values for the Regression Model of TMMRF.

**TABLE A2** Stepwise linear regression results for the vertical range of the orotropical mountain forest belt (OTMF).

| Model | Unstandardized Coefficients | | Standardized Coefficients | t | Sig. | Collinearity Statistics | |
| --- | --- | --- | --- | --- | --- | --- | --- |
|  | B | Std. Error | Beta |  |  | Tolerance | VIF |
| (Constant) | -819.906 | 475.937 |  | -1.723 | 0.103 |  |  |
| BIO5 | 57.268 | 20.714 | 0.557 | 2.765 | **0.013** | 1.000 | 1.000 |

**Model fit:** n = 19, R² = 0.310, F (1, 17) = 7.643, p = **0.013**, significant at p < 0.05 (bold)


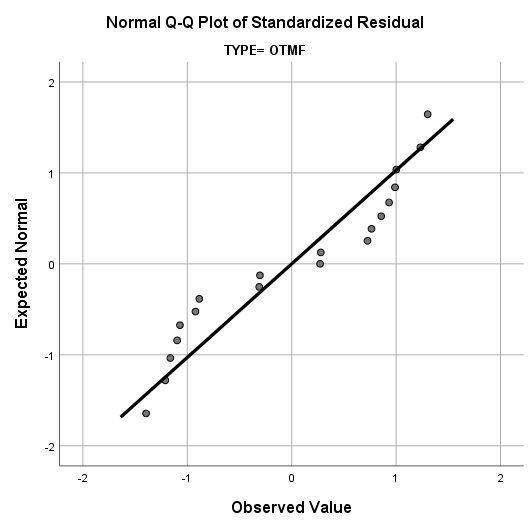


**FIGURE A3** Normal Q-Q Plot of Standardized Residuals for the Regression Model of OTMF.


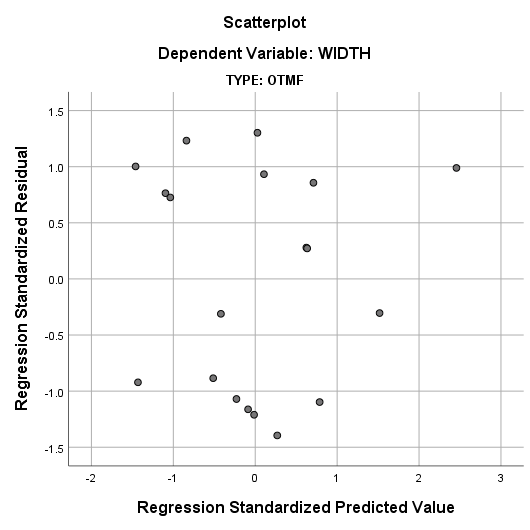


**FIGURE A4** Scatter plot of Standardized Residuals versus Standardized Predicted Values for the Regression Model of OTMF.

**TABLE A3 Stepwise linear regression results for the vertical range of the orotropical bamboo forest belt (OTBF).**

| Model | Unstandardized Coefficients | | Standardized Coefficients | t | Sig. | Collinearity Statistics | |
| --- | --- | --- | --- | --- | --- | --- | --- |
|  | B | Std. Error | Beta |  |  | Tolerance | VIF |
| (Constant) | 116.508 | 109.222 |  | 1.067 | 0.309 |  |  |
| BIO17 | 1.810 | 0.661 | 0.637 | 2.740 | **0.019** | 1.000 | 1.000 |

**Model fit:** n = 13, R² = 0.406, F (1, 11) = 7.507, p = **0.019**, significant at p < 0.05 (bold)


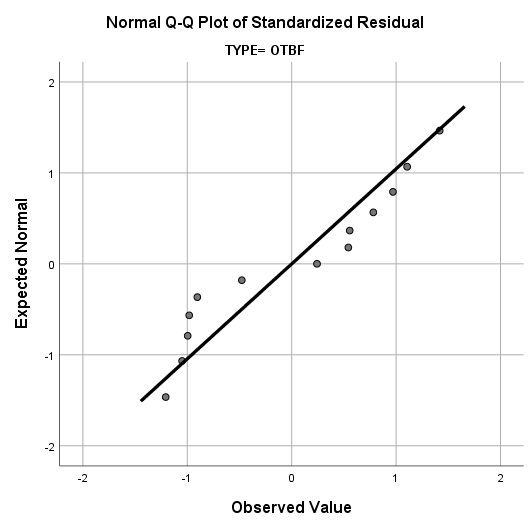


**FIGURE A5** Normal Q-Q Plot of Standardized Residuals for the Regression Model of OTBF.


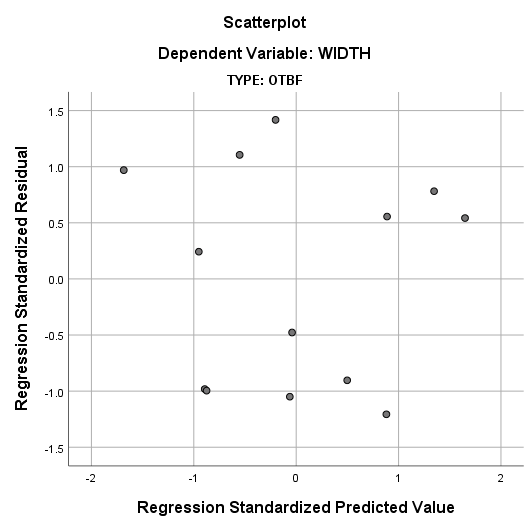


**FIGURE A6** Scatter plot of Standardized Residuals versus Standardized Predicted Values for the Regression Model of OTBF.

**TABLE A4** Stepwise linear regression results for the vertical range of the orotropical cloud forest belt (OTCF).

| Model | Unstandardized Coefficients | | Standardized Coefficients | t | Sig. | Collinearity Statistics | |
| --- | --- | --- | --- | --- | --- | --- | --- |
|  | B | Std. Error | Beta |  |  | Tolerance | VIF |
| (Constant) | -67.966 | 145.726 |  | -0.466 | 0.652 |  |  |
| BIO15 | 5.975 | 2.244 | 0.664 | 2.663 | **0.026** | 1.000 | 1.000 |

**Model fit:** n = 11, R² = 0.441, F (1, 9) = 7.090, p = **0.026**, significant at p < 0.05 (bold)


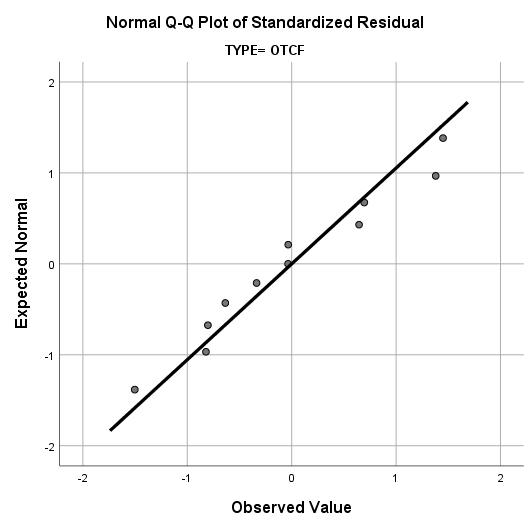


**FIGURE A7** Normal Q-Q Plot of Standardized Residuals for the Regression Model of OTCF.


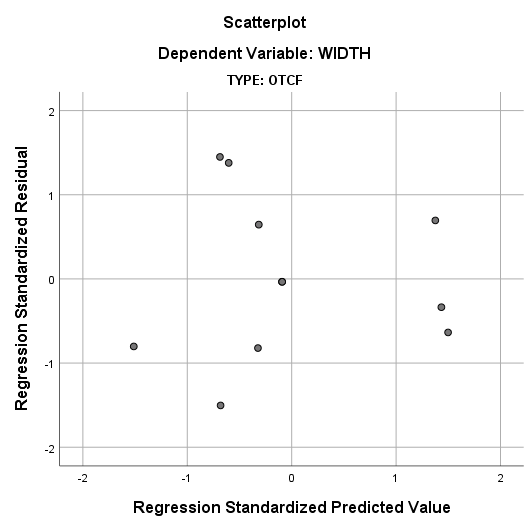


**FIGURE A8** Scatter plot of Standardized Residuals versus Standardized Predicted Values for the Regression Model of OTCF.

**TABLE A5** Stepwise linear regression results for the vertical range of the orotropical ericaceous forest belt (OTEF).

| Model | Unstandardized Coefficients | | Standardized Coefficients | t | Sig. | Collinearity Statistics | |
| --- | --- | --- | --- | --- | --- | --- | --- |
|  | B | Std. Error | Beta |  |  | Tolerance | VIF |
| (Constant) | 3806.070 | 680.213 |  | 5.595 | **0.000** |  |  |
| BIO7 | -125.299 | 22.964 | -0.948 | -5.456 | **0.000** | 0.818 | 1.222 |
| BIO16 | -2.737 | 0.883 | -0.538 | -3.100 | **0.011** | 0.818 | 1.222 |

**Model fit:** n = 13, R² = 0.753, F (2, 10) = 15.253, p = **0.001**, significant at p < 0.05 (bold)


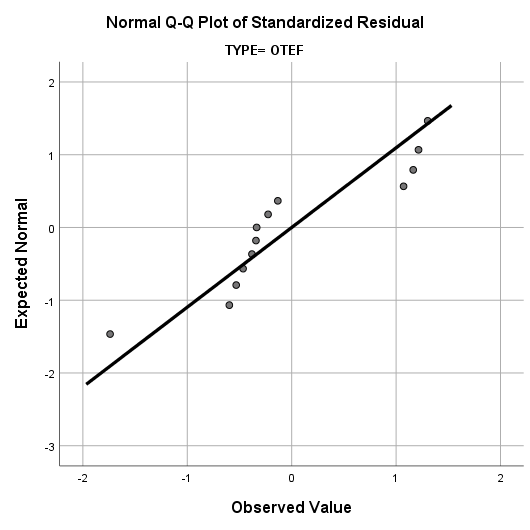


**FIGURE A9** Normal Q-Q Plot of Standardized Residuals for the Regression Model of OTEF.


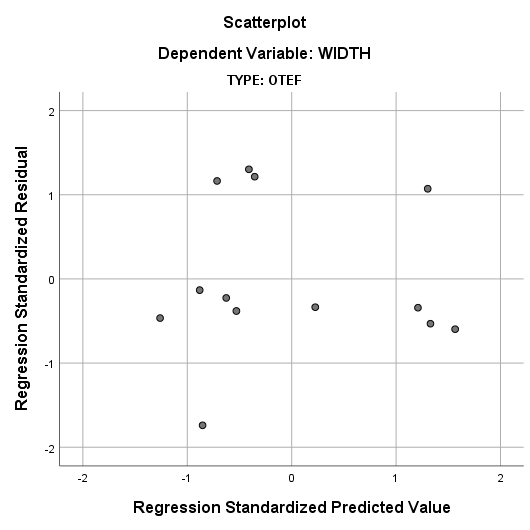


**FIGURE A10** Scatter plot of Standardized Residuals versus Standardized Predicted Values for the Regression Model of OTEF.
